# A Rare Presentation of Indirect Hyperbilirubinemia: Coexistence of Multiple *UGT1A1* Gene Variants

**DOI:** 10.14309/crj.0000000000001436

**Published:** 2024-07-17

**Authors:** Phi Tran, Andrea Grimbergen, Megan Lewis, Ruiyang Yi, Christopher Williams

**Affiliations:** 1 Department of Internal Medicine, Baylor Scott and White Medical Center, Temple, TX; 2 Division of Gastroenterology, Baylor Scott and White Medical Center, Temple, TX; 3 Baylor College of Medicine, Temple, TX; 4 Department of Molecular and Human Genetics, Baylor College of Medicine, Houston, TX

**Keywords:** Crigler-Najjar, Gilbert Syndrome, genetics, hyperbilirubinemia

## Abstract

Indirect hyperbilirubinemia is a common clinical finding and rarely can be attributed to Crigler-Najjar syndrome type 2 or Gilbert syndrome. This case displays a rare presentation of indirect hyperbilirubinemia in a patient with multiple *UGT1A1* gene variants. We aim to discuss the complexity of multiple *UGT1A1* gene variants and its effect on the degree of observed hyperbilirubinemia.

## INTRODUCTION

Crigler-Najjar syndrome (CNS) type 2 is a rare genetic disorder with an incidence of less than 1 in 1 million people around the world.^1^ Gilbert syndrome (GS) is the most common hereditary disorder of bilirubin glucuronidation, known to affect 3%–8% of the global population.^1–4^ Both are characterized by nonhemolytic unconjugated hyperbilirubinemia because of mutations in the uridine 5'-diphospho-glucuronosyltransferase family 1 member A1 gene (*UGT1A1*).^2^ CNS type 2 is an autosomal recessive disorder caused by missense mutations within the *UGT1A1* coding region, resulting in amino acid substitutions that markedly reduce the catalytic activity of *UGT1A1* to less than 10% of normal enzyme activity.^5^ GS is an autosomal recessive disorder caused by a homozygous insertion of a TA dinucleotide in the TATA element of the promoter region of the *UGT1A1* gene.^6,7^ This variant is termed UGT1A1*28 and typically results in reduced catalytic activity of *UGT1A1* to less than 30% of normal enzyme activity.^5^ Loss of bilirubin uridine diphosphate-glucuronosyltransferase enzyme function decreases glucuronidation of unconjugated bilirubin and leads to a toxic accumulation of unconjugated bilirubin, resulting in indirect hyperbilirubinemia and jaundice. In both CNS type 2 and GS, patients typically present with asymptomatic jaundice and isolated indirect hyperbilirubinemia, although average unconjugated bilirubin levels are often higher in CNS Type 2 (6–20 mg/dL) than in GS (1–5 mg/dL).^2,4^ Genetic testing of *UGT1A1* gene for mutations is diagnostic, although rarely reported. Treatment is typically supportive. Genetic counseling may be beneficial. Monitoring for symptomatic triggers may be beneficial for preventing bilirubin-induced injuries. This case describes a unique presentation of multiple *UGT1A1* gene variants, resulting in asymptomatic indirect hyperbilirubinemia.

## CASE REPORT

A 20-year-old man presented with 2.5-year history of intermittent scleral icterus with indirect hyperbilirubinemia, ranging from 1.8 to 7.2 mg/dL. He denied jaundice, abdominal pain, abdominal swelling, lower extremity swelling, melena, hematemesis, fevers, chills, confusion, tremor, rash, or altered sleep-wake cycle. His vitals, laboratory test results, and physical examination were unremarkable. He had no history of tobacco, alcohol, or illicit drug use. He reported an active lifestyle, no herbal or supplement use, and no current prescription medications. His birth history was uneventful and without a history of neonatal jaundice; he reached all expected developmental milestones. Ceruloplasmin, peripheral blood smear, lactate dehydrogenase, haptoglobin, acetaminophen levels, and thyroid-stimulating hormone were all within normal limits. Acute viral hepatitis serologies were as follows: negative hepatitis A virus immunoglobulin M (IgM), negative hepatitis B core IgM, negative hepatitis B surface antigen, and negative hepatitis C antibody by qualitative chemiluminescent immunoassay. An abdominal ultrasound revealed normal liver size (15.4 cm), morphology, and echogenicity without intrahepatic biliary dilatation. The gallbladder was unremarkable and without stones or pericholecystic fluid. Given the patient's asymptomatic indirect hyperbilirubinemia, the differential included CNS type 2 vs GS, and the patient was sent for targeted mutation analysis for *UGTA1A1* clinically significant variants. Genetic testing demonstrated 2 variants, UGT1A1:NM_000463.2:c.674T>G het p.(Val225Gly), as well as UGT1A1:NM_000463.3:c.-41_-40dupTA hom. The patient followed up with the genetics department for further counseling. He was advised to stay well hydrated and educated about the risks of worsening hyperbilirubinemia during fasting or illness and the importance of evaluating serum bilirubin during periods of illness or worsening jaundice. He was also given reproductive counseling regarding the risks of passing along his *UGT1A1* gene variants to future children.

## DISCUSSION

This patient had a combined heterozygous *UGT1A1* coding variant with a homozygous UGT1A1*28 polymorphism. The homozygous UGT1A1*28 polymorphism is often associated with GS, and similarly to GS patients, our patient had reduced UGT1A1 activity without complete cessation of enzyme activity.^8^ With the additional heterozygous *UGT1A1* coding variant, we suspect that his intermediate degree of hyperbilirubinemia, between the reported average ranges for both CNS type 2 and GS, may reflect the cumulative effects of multiple *UGT1A1* gene variants present. Previous case reports have demonstrated that the coexistence of multiple *UGT1A1* gene variants can cause a cumulative effect on the degree of hyperbilirubinemia by reducing expression of functional *UGT1A1* enzyme.^7,9^

Maruo et al described a patient with 3 variants including a c.381insGG frameshift change often associated with CNS type 1, a c.674T>G (p. V225G) more often associated with CNS type 2 and a promoter variant A(TA)7TAA more commonly associated with GS.^9^ Please note that the degree of *UGT1A1* enzyme expression and resulting hyperbilirubinemia likely depends on whether the frameshift change was in cis or trans with other changes. We suspect the patient in Maruo's case had a frameshift change that was in trans with other changes, allowing for a completely nonfunctional copy of the gene (the frameshift change) and another that was partially defective (the point mutation and promoter sequence change), which would create a more severe presentation that our patient's own presentation.

Historically, CNS type 2 and GS were believed of as syndromes with distinct genotype and phenotype. However, recent studies have identified patients with combinations of genetic polymorphisms classically associated with CNS type 2 and GS, respectively, who demonstrate degrees of hyperbilirubinemia between that of CNS type 2 and GS.^5–7^ A previous case report described a young adult with heterozygous mutations for CNS type 2 and homozygous mutations for GS who developed kernicterus after a laparoscopic cholecystectomy.^10^ The authors noted how combinations of benign defects could cumulate in clinically severe diseases. Other cases describe episodes of severe hyperbilirubinemia, leading to acute bilirubin encephalopathy and kernicterus.^11,12^ In addition, more than half of the Western population may be heterozygous carriers of the Gilbert-type promoter gene.^2^ We suspect that some patients may have combined *UGT1A1* polymorphisms with 1 heterozygous missense allele and another heterozygous promoter variant in the other allele. These combined defects may explain intermediate levels of hyperbilirubinemia in family members of patients with both CNS types 1 and 2.

Given the cumulative effect of multiple defects leading to variable degrees of hyperbilirubinemia, as well as the prevalence of carrier genes, genetic counseling for possibilities of inherited jaundice and kernicterus in newborns should be emphasized.^4^ Treatment and interventions are typically supportive; however, early identification and monitoring remain essential for preventing bilirubin-related injury.

For patients presenting with asymptomatic indirect hyperbilirubinemia in the absence of clear etiology, testing for *UGT1A1* gene variants should be considered. Heterozygous *UGT1A1* gene variants, CNS type 2, and GS may be more of a spectrum rather than isolated syndromes as classically taught. They may be more common than previously perceived. Limitations of this case presentation include small sample sizes, and case reports regarding genetic presentations of elevated indirect hyperbilirubinemia are sparse. Even more sparse are articles explaining the phenotypic overlap between GS and CNS type 2. This case highlights the clinical importance of this spectrum because patients will typically have higher indirect bilirubin than single-syndrome patients, and there is evidence of the cumulative effect of hyperbilirubinemia in this population.

## DISCLOSURES

Author contributions: P. Tran: wrote case presentation and discussion; A. Grimbergen: wrote introduction and assisted with discussion; M. Lewis: reviewed the case and addressed reviewer comments; R. Yi: assisted with reviewer comments regarding genetics specific comments; C. Williams: assisted with finalizing case report and addressing reviewer comments. All authors agree to be accountable for the final version. P. Tran is the article guarantor.

Financial disclosure: None to report.

Informed consent was obtained for this case report.
